# Deep learning-aided respiratory motion compensation in PET/CT: addressing motion induced resolution loss, attenuation correction artifacts and PET-CT misalignment

**DOI:** 10.1007/s00259-024-06872-x

**Published:** 2024-08-13

**Authors:** Yihuan Lu, Fei Kang, Duo Zhang, Yue Li, Hao Liu, Chen Sun, Hao Zeng, Lei Shi, Yumo Zhao, Jing Wang

**Affiliations:** 1 United Imaging Healthcare, No. 2258 Chengbei Road, Shanghai, 201807 China; 2 Department of Nuclear Medicine, Xijing Hospital, Fourth Military Medical University, 127 West Changle Road, Xi’an, 710032 China

**Keywords:** PET, Data-driven, Respiratory motion correction, uExcel OncoFocus, Deep learning, uMI Panorama

## Abstract

**Purpose:**

Respiratory motion (RM) significantly impacts image quality in thoracoabdominal PET/CT imaging. This study introduces a unified data-driven respiratory motion correction (uRMC) method, utilizing deep learning neural networks, to solve all the major issues caused by RM, i.e., PET resolution loss, attenuation correction artifacts, and PET-CT misalignment.

**Methods:**

In a retrospective study, 737 patients underwent [^18^F]FDG PET/CT scans using the uMI Panorama PET/CT scanner. Ninety-nine patients, who also had respiration monitoring device (VSM), formed the validation set. The remaining data of the 638 patients were used to train neural networks used in the uRMC. The uRMC primarily consists of three key components: (1) data-driven respiratory signal extraction, (2) attenuation map generation, and (3) PET-CT alignment. SUV metrics were calculated within 906 lesions for three approaches, i.e., data-driven uRMC (proposed), VSM-based uRMC, and OSEM without motion correction (NMC). RM magnitude of major organs were estimated.

**Results:**

uRMC enhanced diagnostic capabilities by revealing previously undetected lesions, sharpening lesion contours, increasing SUV values, and improving PET-CT alignment. Compared to NMC, uRMC showed increases of 10% and 17% in SUV_max_ and SUV_mean_ across 906 lesions. Sub-group analysis showed significant SUV increases in small and medium-sized lesions with uRMC. Minor differences were found between VSM-based and data-driven uRMC methods, with the SUV_max_ was found statistically marginal significant or insignificant between the two methods. The study observed varied motion amplitudes in major organs, typically ranging from 10 to 20 mm.

**Conclusion:**

A data-driven solution for respiratory motion in PET/CT has been developed, validated and evaluated. To the best of our knowledge, this is the first unified solution that compensates for the motion blur within PET, the attenuation mismatch artifacts caused by PET-CT misalignment, and the misalignment between PET and CT.

**Supplementary Information:**

The online version contains supplementary material available at 10.1007/s00259-024-06872-x.

## Introduction

The clinical and research applications of PET/CT have expanded significantly, owing to advancements in hardware such as silicon photomultipliers and time-of-flight (TOF) techniques, as well as in software such as novel image reconstruction algorithms [1, 2]. These improvements have empowered PET/CT systems to deliver enhanced spatial and temporal resolution [3–5], coupled with improved sensitivity [6]. Nevertheless, these advancements contrast with persistent challenges in thoracoabdominal PET/CT imaging, primarily attributable to respiratory motion (RM). RM stands out as a predominant factor in PET imaging artifacts, notably inducing resolution loss due to motion, PET-CT misalignment induced artifacts in attenuation correction (AC), and inaccuracies in the localization of lesions or organs in PET/CT fusion images. Studies have illustrated that ungated PET/CT examinations near the diaphragm can lead to blurred lesion margins, artificially decreased Standard Uptake Values (SUV), inaccuracies in lesion localization, and the presentation of lesions as more extensive yet less distinct.

In addressing RM artifacts, various strategies have been explored, including prone positioning [7], rescanning, breath-hold techniques [8], 4D-CT [9], device-based [10], and data-driven respiratory gating [11]. Recently, deep learning (DL)-based techniques have gained widespread adoption in PET imaging and reconstruction [12, 13]. Specifically, for mitigating motion-induced artifacts in PET, several DL-based methods have been proposed, for instance, to enhance motion estimation and provide more precise attenuation correction [14–18]. Among these efforts, one approach involves combining time-of-flight (TOF) capabilities with simultaneously estimating emission and transmission images [19, 20] and DL techniques to address attenuation correction artifacts caused by PET-CT mismatches [17, 21, 22]. Compared to traditional methods that simultaneously estimate emission and transmission images, using DL techniques to further enhance the attenuation map estimated by conventional methods results in higher quality images, thereby improving the accuracy of PET attenuation correction [21, 22]. Despite significant progress, integrating these methods into routine clinical workflows remains challenging, often leading to increased operational and reconstruction times. More importantly, these techniques do not fully overcome PET-CT mis-registration, thus limiting their clinical utility.

In this study, we introduce a unified data-driven solution for respiratory motion correction (uRMC), marketed under the name uExcel OncoFocus (United Imaging Healthcare, Shanghai, China). With the aid of deep learning neural networks, uRMC is designed to address RM induced challenges in PET/CT, specifically mitigating motion blur within PET, preventing AC artifacts caused by PET-CT misalignment, and aligning PET-CT in the final images, without any external device. In addition, we present the RM magnitude of major organs.

## Materials and methods

### Human subjects and data acquisitions

In this retrospective single-center study, we examined 737 patients (Table 1) who underwent whole-body [^18^F]FDG PET/CT scans (60 min post-injection of 276 ± 80 MBq) using the United Imaging uMI Panorama PET/CT scanner at Xijing Hospital, Xi’an, China. All the PET scans were conducted a ‘neck-to-thigh’ scan, resulting in 4 bed positions with 2 min scan duration for each bed position. The study, conducted from September 2021 to October 2022, received ethical approval from the institutional review board of Xijing Hospital (Approval No. KY-20212145-F-1). Given the retrospective nature of the study, informed consent was waived. The dataset was bifurcated, with ninety-nine patients undergoing PET/CT scans coupled with VSM (vital signal module, a RM monitoring device) [23] data acquisition, constituting the *validation dataset*. The remaining 638 patients had their images utilized for neural networks training in the uRMC development.

**Table 1:** Demographic information of the patient datasets

Patient sets	Characteristic		Mean ± SD	Number
Training set				
	Patient number			638
	Male			394
	Female			244
	Age (y)		56.0 ± 16.0	
	Height (cm)		166.0 ± 10.2	
	Weight (kg)		63.6 ± 13.9	
	BMI		22.9 ± 4.0	
	Injected dose (MBq)		280.1 ± 86.2	
Validation set				
	Patient number			99
	Male			54
	Female			45
	Age (y)		58.4 ± 15.5	
	Height (cm)		166.0 ± 8.1	
	Weight (kg)		64.8 ± 12.2	
	BMI		23.3 ± 3.7	
	Injected dose (MBq)		252.7 ± 48.8	
	Number of disease cases			
		Lung cancer		29
		Lymphoma, leukemia and myeloma		17
		Liver cancer		8
		Colorectal cancer		7
		Gastric cancer		5
		Cervical carcinoma		5
		Pancreatic cancer		5
		Bone and soft tissue sarcoma		3
		Others		20

SD: standard deviation

### Algorithm overview

**Fig. 1:**
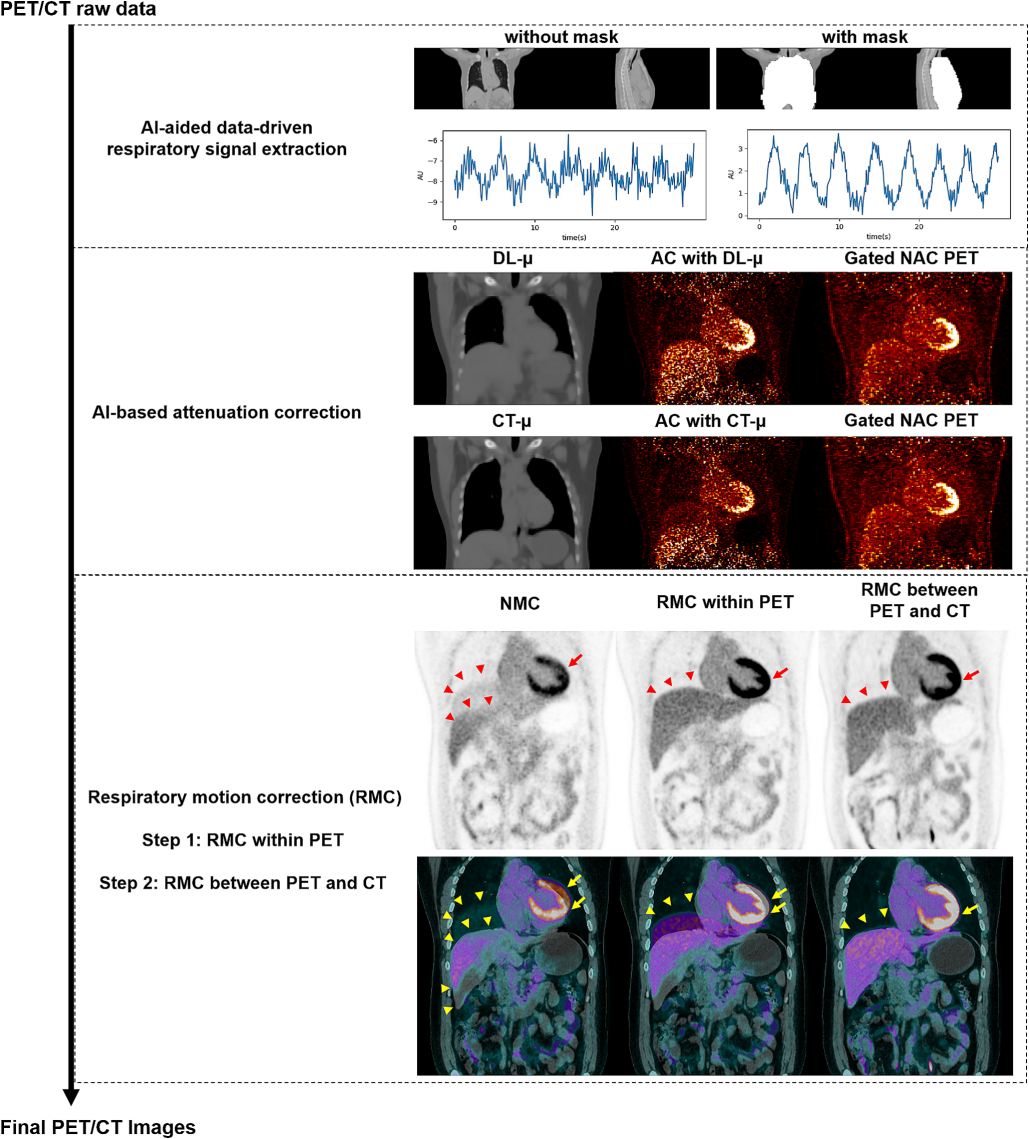
The proposed uRMC framework. AI: artificial intelligence; DL: deep learning; NAC: without attenuation correction; NMC: no motion correction; uRMC: unified respiratory motion correction

The proposed uRMC primarily consists of three key components: (1) respiratory signal extraction, (2) attenuation map generation, and (3) PET-CT alignment. Leveraging two deep learning neural networks, one data-driven RM detection algorithm, and an anatomy-based non-rigid image registration algorithm, uRMC mitigates artifacts arising from RM in PET/CT. The overarching framework design of uRMC is depicted in Fig. 1.

### AI-aided data-driven respiratory signal extraction

In light of respiratory physiology, we posit that annihilation events within the thoracic, abdominopelvic cavities, and the surrounding anterolateral thoracic and abdominal wall regions exclusively contain relevant respiratory signals; events originating elsewhere can be regarded as noise in the generation of respiratory signals. Consequently, we employed a convolutional neural network (CNN_cavity_), which was trained by a customized 3D U-net with a residual unit network on 160 whole-body CT volume images, to meticulously segment the thoracic and abdominopelvic region of interest (ROI, referred to as the *body cavity*). Subsequently, dorsoventral dilation (50 mm in the anterior-posterior direction) was applied to create the respiratory region of interest (rROI) for extraction of respiratory signals. Details of the body cavity segmentation are provided in the ‘*Body cavity segmentation*’ section of the Supplemental materials.

The centroid-of-distribution (COD) technique [24] was employed for respiratory signal detection. In COD, for every list mode event, the line-of-response (LOR) is determined by the pair of detectors. The spatial coordinates of the two detectors are recorded in millimeters from the center of the field-of-view (FOV). With TOF information available, the center of the TOF bin for each event can be determined along the event LOR. At intervals of 100 milliseconds, the coordinates of all events within the rROI were averaged to generate the COD for that time period. This process was repeated for every 100 msec of the entire PET study, resulting in a 3-D trace denoted as *C*_x_, *C*_y_, and *C*_z_. These represented the lateral (X), anterior-posterior (Y), and superior-inferior (Z) direction COD signals, respectively. In this study, *C*_y_ was specifically utilized for respiratory tracking signal. Based on *C*_y_, motion amplitude-based equal-count gating [25] was implemented to rebin the PET raw data into four respiratory gates. Image reconstruction without AC (NAC) was subsequently performed to obtain the NAC image for each gate (*g*), denoted as ${\rm{\lambda }}_{{\rm{NAC}}}^g$. Figure 1 shows an example of the respiratory extracted signal with and without rROI applied. Supplemental Figs. 3 and 4 in Supplementary materials demonstrate two examples of the COD signal for motion tracking.

### AI-based attenuation correction and motion correction in PET

Another deep learning (DL) network (CNN_mu_, a customized 3D U-net with a residual unit) was trained to predict the gated attenuation map (${\rm{\mu }}_{{\rm{DL}}}^g$) utilizing ${\rm{\lambda }}_{{\rm{NAC}}}^g$ as the network input. The training details of CNN_mu_ is provided in the ‘*AI-based attenuation correction*’ section of the Supplemental materials. It is noteworthy that ${\rm{\mu }}_{{\rm{DL}}}^g$ inherently corresponded to ${\rm{\lambda }}_{{\rm{NAC}}}^g$ in spatial alignment. For each respiratory gate, Ordered Subset Expectation Maximization (OSEM) was applied using ${\rm{\mu }}_{{\rm{DL}}}^g$ for AC. Subsequently, a non-rigid image registration was executed between other gates and the reference gate, which was the gate closest to the CT in terms of respiratory phase, quantified using mutual information between${\rm{\mu }}_{{\rm{DL}}}^g$ and the CT attenuation map. The resultant displacement fields were applied to warp each gated PET image to the reference gate location. The outcome of this registration process, denoted as ${\rm{\lambda }}_{{\rm{RMC}}}$, is achieved by consolidating all warped gated PET images, representing the respiratory motion correction (RMC) result prior to matching with CT. Note that if the CT was acquired in between expiration and inspiration in PET, ${\rm{\lambda }}_{{\rm{RMC}}}$ should match CT. Conversely, if the CT was obtained outside the typical RM range in PET, such as during deep inspiration, PET-CT misalignment will persist even in the absence of RM and AC artifacts in the PET itself, i.e., ${\rm{\lambda }}_{{\rm{RMC}}}$.

### PET-CT alignment

To address the potential misalignment between ${\rm{\lambda }}_{{\rm{RMC}}}$ and the CT, a conventional approach involving multi-modal image registration [18] may be used. It is well known that multi-modal image registration is a challenging task in the computer vision community. In uRMC, we opted for a different strategy to mitigate this challenge. Instead of framing it as a multi-modal registration problem, we transformed the problem into a single-modal image registration. More specifically, we applied non-rigid image registration between the ${\rm{\mu }}_{{\rm{DL}}}^g$ at the reference gate and the CT attenuation map. The resulting displacement field was then utilized to deform ${\rm{\lambda }}_{{\rm{RMC}}}$, ensuring alignment with the CT. The ultimate outcome, denoted as ${\rm{\lambda }}_{{\rm{uRMC}}}$, represented the final RMC image in the CT space.

### Evaluation

For the validation dataset, we conducted two separate PET image reconstructions (${\rm{\lambda }}_{{\rm{uRMC}}}$). These reconstructions utilized the uRMC approach, one with the data-driven respiratory signal, i.e., COD-based [24], and the other using the device-based signal, i.e., VSM [23]. COD-uRMC and VSM-uRMC are identical in data processing, except for the source of the respiratory signals they use, i.e., VSM-uRMC does not require the use of CNN_cavity_ network for body cavity segmentation to achieve data-driven respiratory signal extraction. These reconstructions were then compared with the standard OSEM reconstructions that did not incorporate motion correction (NMC). This comparative analysis aimed to evaluate not only the effectiveness of the uRMC but also the overall robustness of the proposed data-driven approach. The assessment specifically focused on the lesion ROI level.

Lesion ROIs were delineated using another U-net segmentation neural network (CNN_lesion_) trained on a publicly available whole-body [^18^F]FDG PET/CT dataset [26] with manually annotated lesions. CNN_lesion_ was applied to the VSM-based ${\rm{\lambda }}_{{\rm{uRMC}}}$ to segment potential lesions, thus creating lesion ROIs. SUV metrics, including SUV_mean_ and SUV_max_, were calculated within these ROIs for all three approaches (NMC, VSM-based uRMC and COD-based uRMC). The lesions were categorized based on volume into small (0.1-5.0 mL), medium (5.0–10.0 mL), and large (> 10.0 mL) groups. Average SUV values were calculated for each group and approach, with paired t-tests used to evaluate significant differences between methods.

We also present the motion amplitudes of various organs within the validation set. The process for quantifying motion amplitude involves several steps: initially, TotalSegmentator [27] was used to delineate organ ROIs on CT images. These organs included the aortic arch, heart, lungs, ribs, chest wall, liver, spleen, stomach, pancreas, kidneys, colon, bladder, and abdominal wall. Then, these organ ROIs were resliced using the inverse of the PET-CT alignment displacement field to align them with the PET reference gate. The average of the maximal displacement field, which represents the amplitude of motion between the inspiration and expiration phases, was calculated within the ROI of a deformed organ. This was done by determining the maximal displacement for each individual and then taking the average across all patients.

## Results

### Demo cases

**Fig. 2:**
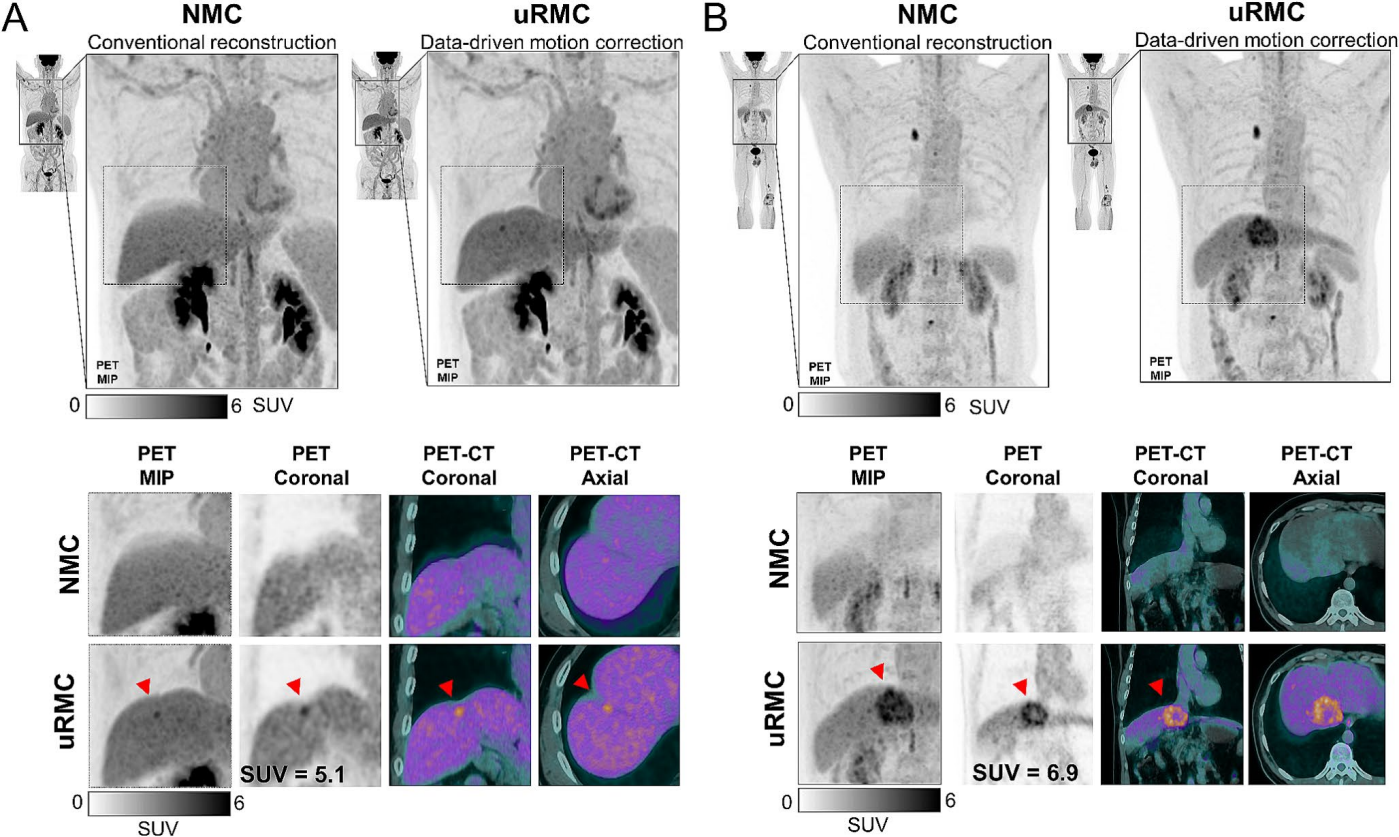
Examples showcasing potential additional diagnostic benefits provided by uRMC. (**A**) In restaging a patient with hepatocellular carcinoma after surgical resection of the primary site, an [^18^F]FDG-avid hepatic lesion is revealed at the resection margin with uRMC, whereas a conventional NMC image shows a negative result. (**B**) During restaging a patient with a prior history of left knee osteosarcoma, CT was acquired at deep inspiration, which mismatches with the PET that was acquired with free-breathing. Results in attenuation artifacts presented as a “banana artifacts” at the liver dome in the NMC images. With the use of uRMC, an annular [^18^F]FDG-avid lesion was unveiled, indicating a metastatic lesion with necrosis. NMC: no motion correction; uRMC: unified respiratory motion correction

**Fig. 3:**
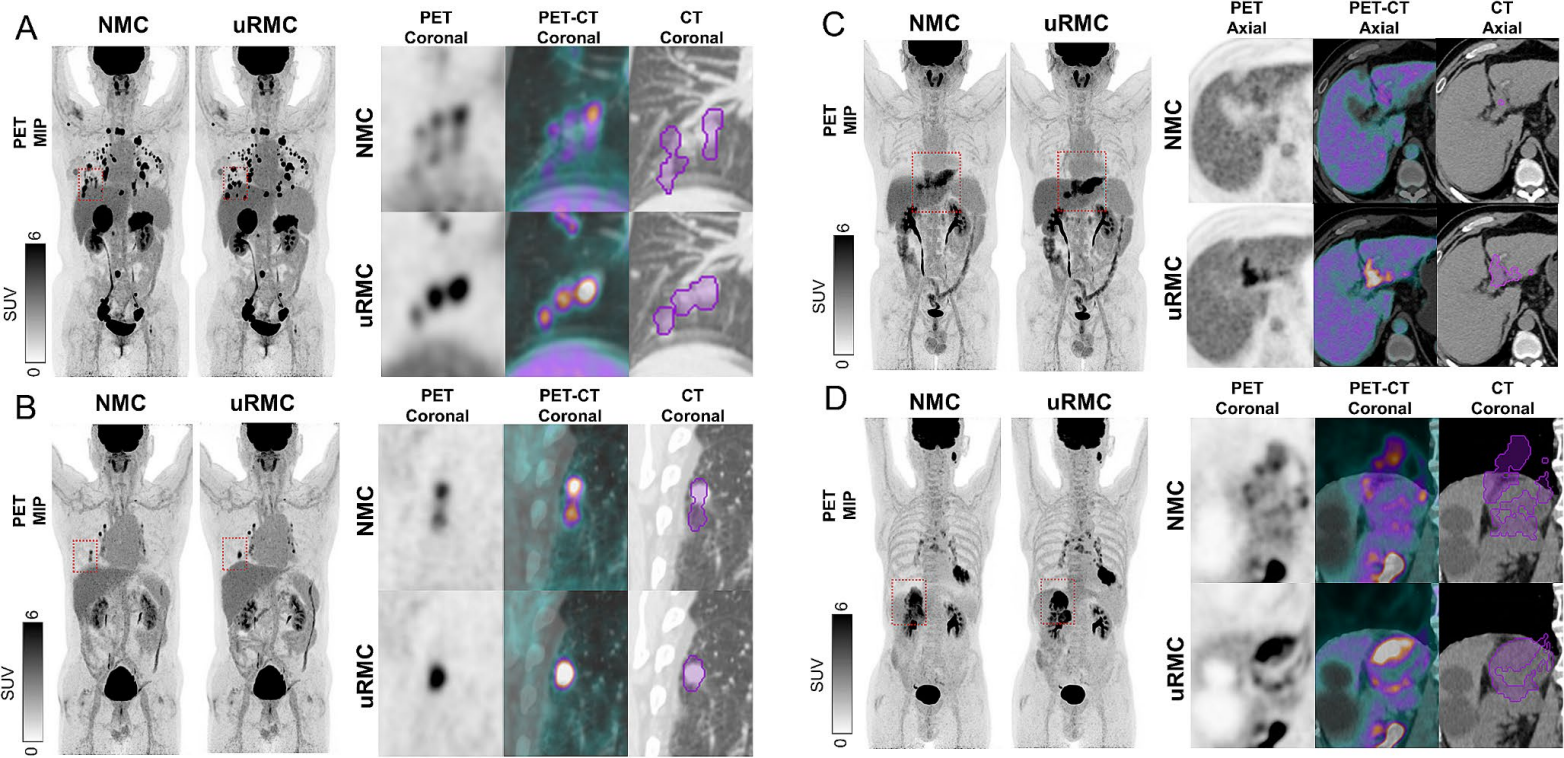
Comparison of NMC with uRMC images. Compared with NMC images, uRMC images show sharper lesion contours, smaller metabolic volume, increase in SUV values, and improved PET-CT alignment. (**A**) Delineation of multiple lower lung nodules in a patient with diffuse metastatic small cell neuroendocrine carcinoma of the cervix. (**B**) Double contouring of a lower lung nodule in the NMC images, whereas uRMC images reveal a single nodular contouring. (**C**) In a patient with hepatocellular carcinoma, an [^18^F]FDG-avid tumor thrombus is clearly seen in the PET and well registered to the portal vein on CT after MC. (**D**) In a patient with lung cancer, an [^18^F]FDG-avid adrenal metastatic mass is well registered in PET/CT after applying uRMC while NMC images exhibit smeared adrenal uptakes due to respiratory motion. NMC: no motion correction; uRMC: unified respiratory motion correction

**Fig. 4:**
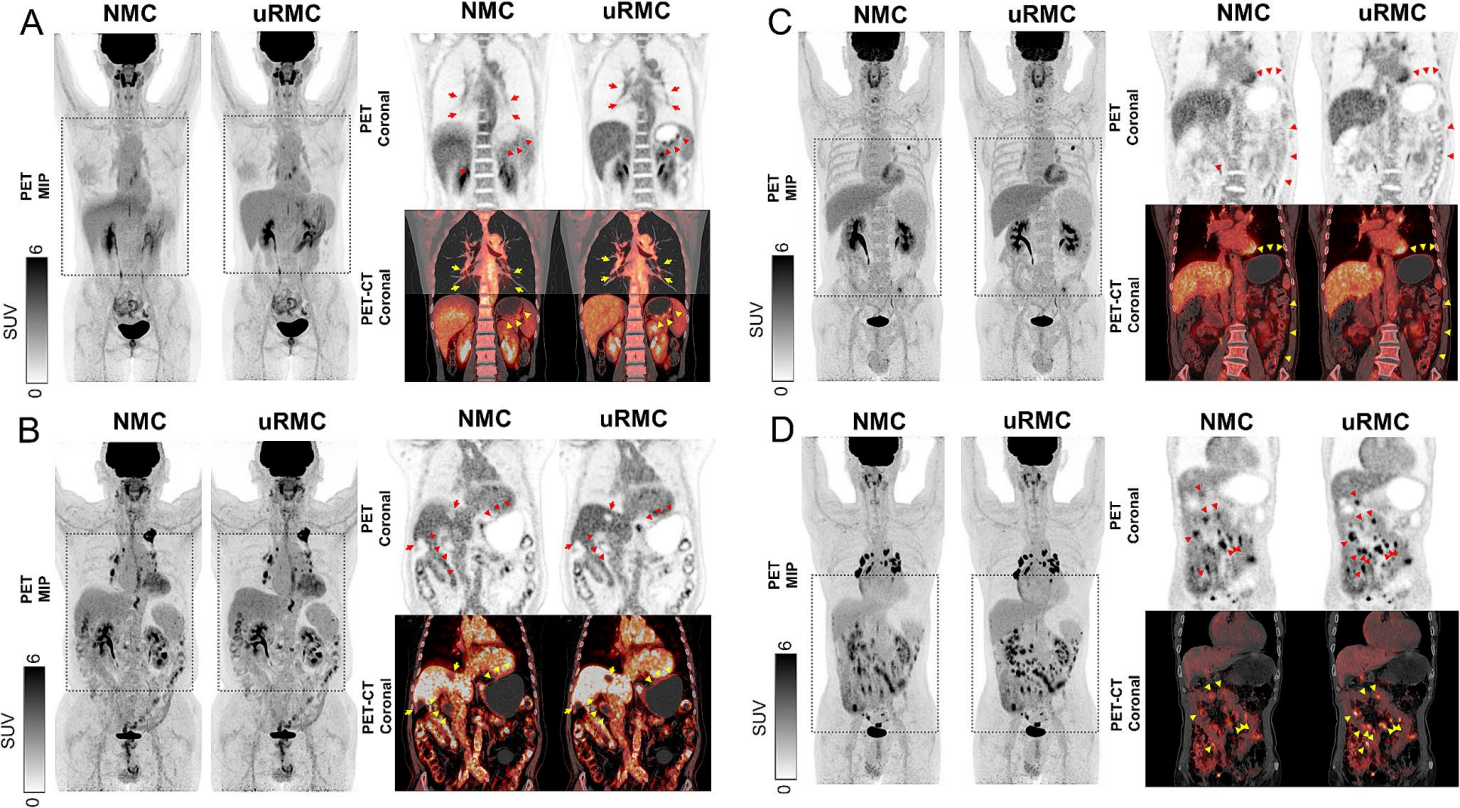
Demonstrating PET delineation and PET-CT alignment differences between NMC and uRMC images for physiological and/or benign uptakes. (**A**) Bronchovascular tree, gastric folds, and hepatosplenic margin are better delineated in uRMC images comparing to blurred NMC images. (**B**) uRMC images reveal cold regions of liver cysts, uptakes of diaphragm, gastric linings, lower esophageal sphincter. (**C**) Gastric folds and the intestinal lining of the descending colon. (**D**) Mesenteric lymph nodes exhibit improved PET recovery and alignment with CT in uRMC images compared to NMC images. NMC: no motion correction; uRMC: unified respiratory motion correction

**Fig. 5:**
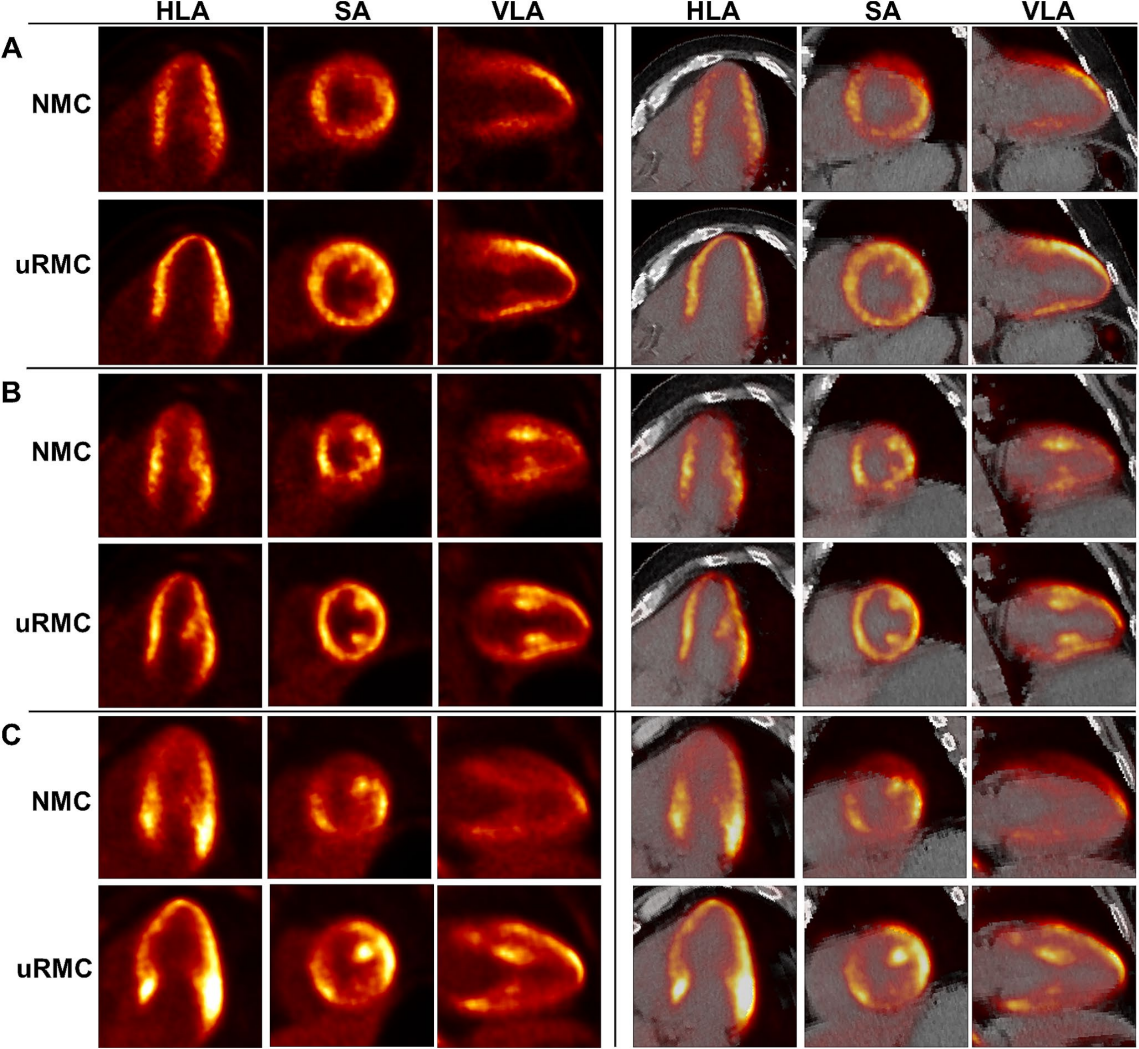
Cardiac PET and PET/CT fusion images from three cases comparing NMC and uRMC images. uRMC shows sharp delineation and PET-CT alignment of ventricular wall and interventricular septal [^18^F]FDG accumulations by eliminating respiratory-motion-induced blurring. NMC: no motion correction; uRMC: unified respiratory motion correction

The potential clinical benefits of the uRMC in comparison to NMC are outlined through Figs. 2, 3, 4 and 5. Figure 2 illustrates the enhanced diagnostic capabilities of uRMC by presenting two clinical cases: one involving restaging of hepatocellular carcinoma post-surgery and another restaging a patient with a history of knee osteosarcoma. In both cases, uRMC reveals previously undetected lesions compared to NMC images. Figure 3 provides a direct comparison between NMC and uRMC images, demonstrating that uRMC results in sharper lesion contours, smaller metabolic volumes, increased SUV values, and improved PET-CT alignment. Examples include cases of lung nodules, tumor thrombus in portal vein, and adrenal metastatic mass in lung cancer. Figure 4 further supports the clinical utility of uRMC by highlighting the delineation and PET-CT alignment differences between NMC and uRMC images for physiological and benign uptakes, such as bronchovascular tree, gastric folds, hepatosplenic margin, liver cysts, diaphragm, gastric linings, and lymph nodes. Additionally, Fig. 5 focuses on cardiac PET and PET/CT fusion images, illustrating the advantages of uRMC in achieving sharp delineation and improved registration of ventricular wall and interventricular septal [^18^F]FDG accumulations by eliminating blurring associated with RM. Overall, the figures collectively emphasize the potential clinical benefits of uRMC over NMC in various diagnostic scenarios. All the demo cases were reconstructed using COD-based uRMC.

### Quantitative analysis

**Fig. 6:**
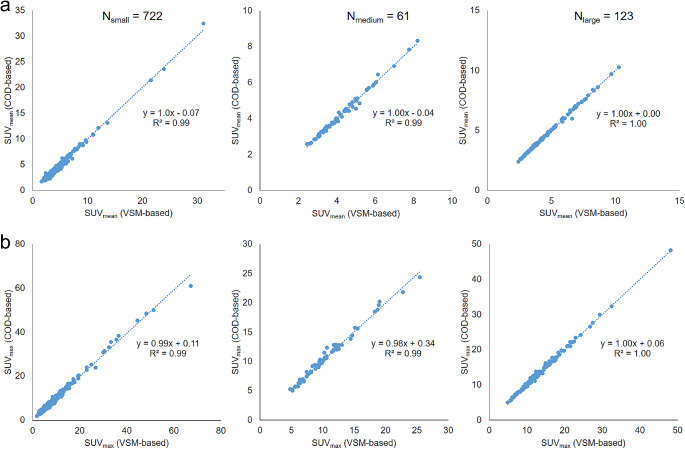
The SUV value comparison results between VSM-based uRMC (gold standard) and data-driven (COD-based) uRMC (proposed). (**a**) Results of SUV_mean_ and (**b**) SUV_max_ for different lesion sizes (small: 0.1-5 mL, *N* = 722; medium: 5–10 mL, *N* = 61; and large: >10 mL, *N* = 123) are presented. Linear correlation analyses of the two methods are shown

For the ninety-nine patients, a total of 906 lesions were segmented for quantitative analysis (see Table 2). This study compared NMC, VSM-based uRMC, and COD-based uRMC. Overall, uRMC showed an approximately increase of 10% and 17% in SUV_max_ and SUV_mean_, respectively, when compared to NMC across all 906 lesions. Sub-group analysis revealed statistically significant increase in SUV_max_ and SUV_mean_ for both small (0.1 mL < volume ≤ 5 mL, *N* = 722) and medium lesions (5 mL < volume ≤ 10 mL, *N* = 61) when using any uRMC method compared to NMC. In the category of large lesions (volume > 10 mL, *N* = 123), a considerable increase in SUV_max_ and SUV_mean_ was observed between NMC and uRMC, although this did not reach statistical significance. Only minor differences were noted between the VSM-based and COD-based uRMC methods. Detailed SUV comparisons for each lesion size category between VSM-based and COD-based uRMC methods are presented in Fig. 6. The comparative analysis of VSM-based and COD-based uRMC revealed negligible differences in SUV_max_ and SUV_mean_, pointing to their similar effectiveness.

**Table 2:** SUV value of lesions with different sizes segmented by the AI network, for the NMC, the device-based uRMC (VSM-uRMC) and the proposed data-driven uRMC (COD-uRMC)

Parameter		NMC	VSM-uRMC	COD-uRMC
SUV_max_				
	**All Lesions (906)** ^**#**^	8.61 ± 13.17	9.49 ± 18.73	9.56 ± 19.69
	P value vs. NMC		0.002	0.002
	P value vs. VSM- uRMC			0.078
	**Small Lesion (0.1–5.0 ml) (722)** ^*^	7.00 ± 7.92	7.75 ± 7.52	7.76 ± 7.64
	P value vs. NMC		< 0.001	< 0.001
	P value vs. VSM- uRMC			0.484
	**Medium Lesion (5.0–10.0 ml) (61)**	9.85 ± 4.18	10.66 ± 4.31	10.78 ± 4.25
	P value vs. NMC		0.001	< 0.001
	P value vs. VSM- uRMC			0.047
	**Large Lesion (> 10.0 ml) (123)**	17.30 ± 28.15	18.95 ± 45.71	19.29 ± 48.4
	P value vs. NMC		0.402	0.364
	P value vs. VSM- uRMC			0.167
**SUV**_**mean**_				
	**All Lesions (906)**	3.52 ± 2.82	4.10 ± 2.78	4.05 ± 2.79
	P value vs. NMC		< 0.001	< 0.001
	P value vs. VSM- uRMC			< 0.001
	**Small Lesion (0.1–5.0 ml) (722)**	3.25 ± 2.31	3.91 ± 2.18	3.85 ± 2.22
	P value vs. NMC		< 0.001	< 0.001
	P value vs. VSM- uRMC			< 0.001
	**Medium Lesion (5.0–10.0 ml) (61)**	3.78 ± 1.24	4.23 ± 1.23	4.21 ± 1.24
	P value vs. NMC		< 0.001	< 0.001
	P value vs. VSM- uRMC			0.176
	**Large Lesion (> 10.0 ml) (123)**	4.90 ± 4.84	5.15 ± 5.12	5.14 ± 5.08
	P value vs. NMC		< 0.001	< 0.001
	P value vs. VSM-uRMC			0.382

^**#**^Lesion category (Number of lesions)

^*^Lesion category (lesion volume) (Number of lesions)

### Motion amplitude measurement for organs

Table 3 presents a summary of the RM amplitude across various organs for each patient. Notably, male patients exhibit greater RM than female patients. Generally, motion amplitudes ranging from 10 to 20 mm are observed in most major organs, with organs nearer to the diaphragm experiencing more significant movement than those further away. For example, the liver displays the highest motion amplitude (22.9 ± 7.6 mm for males and 20.2 ± 4.2 mm for females), which is the most pronounced among all organs, while the bladder shows the least motion (5.9 ± 2.8 mm for males and 4.9 ± 1.5 mm for females). Additionally, the right lung and rib have larger motion amplitudes compared to their left-sided counterparts.

**Table 3:** Comparison of mean of the maximum motion amplitude (in mm) of each organ

Organ	Male (*N* = 54)	Female (*N* = 45)
Aortic arch	7.1 ± 2.2	7.0 ± 1.9
Heart	14.2 ± 4.2	13.7 ± 3.0
Left lung	16.5 ± 4.9	14.4 ± 3.0
Right lung	21.7 ± 7.4	19.6 ± 4.6
Left ribs	11.0 ± 3.8	9.8 ± 2.4
Right ribs	15.3 ± 5.9	15.3 ± 4.7
Chest wall	18.1 ± 6.1	16.9 ± 4.4
Liver	22.9 ± 7.6	20.2 ± 4.2
Spleen	16.0 ± 4.5	13.8 ± 2.9
Stomach	18.0 ± 4.5	16.1 ± 3.1
Pancreas	15.6 ± 4.3	13.5 ± 2.9
Left kidney	15.0 ± 4.6	12.2 ± 2.8
Right kidney	15.3 ± 4.9	13.8 ± 3.4
Colon	17.5 ± 5.4	15.7 ± 3.8
Bladder	5.9 ± 2.8	4.9 ± 1.5
Abdominal wall	17.1 ± 5.5	15.8 ± 3.5

## Discussion

In this study, we proposed a unified data-driven solution for respiratory motion (uRMC) in PET/CT. The proposed solution addressed all the challenges introduced by RM, i.e., mitigating motion blur within PET, preventing attenuation mismatch artifacts caused by PET-CT misalignment as well as aligning PET-CT in the final images. uRMC was validated by comparing the data-driven solution (proposed) to the same method but with device-based respiratory signal tracking (VSM-based). Validation on the ninety-nine [^18^F]FDG clinical whole-body PET/CT studies demonstrated the high robustness of the proposed uRMC approach, even in the presence of metal artifacts or respiratory motion artifacts in the CT images (Supplementary Fig. 5). The proposed method yielded minor differences as compared to the device-based solution in lesion SUV measures (Table 2).

The uRMC was operated on a computer equipped with two Intel(R) Xeon(R) Gold 6134 CPUs and four Nvidia V100 16GB GPUs. When the reconstruction image matrix size was set to 256 × 256, the complete processing for four bed positions was about 9 min.

Traditional PET/CT involves breath coaching, requiring patients to breathe shallowly or hold their breath during acquisition [28–31] and mandates technologists to evaluate respiratory artifacts and associated image quality after the scan. The exclusion of respiration coaching by uRMC offers several advantages, enhancing patient compliance, particularly for those with claustrophobia or critical illness. Operationally, uRMC streamlines PET/CT procedures by reducing breath coaching time, minimizing the need for manual image quality checks and potentially preventing re-scans due to significant motion artifacts. Moreover, the reduced demand for staff training in breathing management contributes to a more versatile workforce, fostering cost savings and optimized staff allocation in healthcare systems.

In RM management techniques, unlike respiratory gating, which only uses partial data for PET reconstruction, RMC method can improve image quality by utilizing all PET counts. RMC could be realized by registering PET images from different gates to a reference gate and summing all images afterward [32, 33], or directly incorporating the calculated image motion information during image reconstruction [14, 20, 34]. Additionally, although respiratory gating can reduce motion-induced blurring artifacts in PET images, misalignment between gated PET images and CT images can still lead to AC artifacts in the final images. In Supplementary Fig. 6, we also present two cases where misalignment between PET and CT resulted in AC artifacts in the respiratory-gated PET images. This issue is also common in most RMC methods, which are unable to address the mis-registration problem between PET and CT. In a previous study [18], PET/CT registration was performed without RMC, which means AC is applied based on the averaged respiratory state and yielded another partial solution. Furthermore, Lu et al. [20] and Hwang et al. [22] used methods to directly obtain attenuation information from the raw PET data, providing precise AC for each respiratory gate. They then used image registration to obtain deformation information between the gated PET images to address motion artifacts in PET images. However, the issue of misalignment between the final PET image and the CT image still remains unresolved. The proposed uRMC essentially addressed a similar problem with the previous study and goes further by solving both problems of RMC and PET-CT registration, which effectively eliminates almost all artifacts caused by RM.

In uRMC, the registration process serves two purposes: (1) correcting respiratory motion by registering PET gated images, and (2) aligning PET/CT by registering ${\rm{\mu }}_{{\rm{DL}}}^g$ with the CT attenuation map. The second part presents additional challenges due to the synthesis step involved. Directly registering ${\rm{\mu }}_{{\rm{DL}}}^g$ from different gates to the CT attenuation map is a simplified approach to achieve both respiratory motion correction and PET/CT alignment simultaneously. However, inconsistencies in ${\rm{\mu }}_{{\rm{DL}}}^g$ generation can reduce the robustness of this direct approach. To achieve better RMC results, we decoupled the gated PET registration from the overall registration process to avoid potential errors associated with the synthesis step.

While previous studies have extensively examined human RM [35–37], this study is unique in reporting the magnitude of motion across various organs. We observed a substantial organ motion ranging from 10 to 20 mm for major organs, which was in stark contrast to the designed spatial resolution of modern PET scanners, such as sub-3 mm resolution, and even finer resolutions down to 1.6 mm achievable with PSF-based resolution recovery for uMI Panorama [3]. This significant discrepancy in orders of magnitude underscores the critical need for RMC in PET/CT imaging to realize the full potential of a PET/CT scanner’s designed spatial resolution. Such correction is essential to improve diagnostic accuracy and the effectiveness of PET/CT in clinical settings.

Repeatability and reproducibility of PET studies are foundational to maintaining the validity of results across studies, ensuring the assessments of disease status, maintaining the consistency of diagnoses, and increasing the diagnostic confidence. For instance, the breathing pattern may change across different PET scans, and their CT may be acquired at different respiratory phase, whereas uRMC can compensate for such differences between PET scans and ensure the PET-CT alignment, potentially benefits multi-modality thoracoabdominal radiomics studies [38, 39].

In this work, we acknowledge certain limitations and outline plans for future research. Currently, uRMC has only been validated with [^18^F]FDG tracers. In the future, we aim to extend our solution to include a variety of other tracers. The patient demographic was relatively homogenous, with limited diversity in BMI and age ranges. Future studies will aim to include a broader spectrum of patients, particularly focusing on diverse age groups like young children and different BMI categories, including obese patients. Additionally, this study was conducted at a single site with a limited Chinese patient population and primarily focused on diseases imaged by [^18^F]FDG. To enhance the comprehensiveness of our findings, it is essential to conduct multi-center studies that incorporate a wider range of diseases, such as various inflammatory conditions, in future research. These steps are crucial to broaden the applicability and validation of uRMC in diverse clinical scenarios.

## Conclusion

A unified data-driven solution for respiratory motion in PET/CT has been developed, validated and evaluated. To the best of our knowledge, this is the first unified solution that compensates for the motion blur within PET, the attenuation mismatch artifacts caused by PET-CT misalignment and the misalignment between PET and CT.

## Electronic supplementary material

Below is the link to the electronic supplementary material.

Supplementary Material 1

## Data Availability

Not applicable.
