# Supplementary material for: Deep learning-aided respiratory motion compensation in PET/CT: addressing motion induced resolution loss, attenuation correction artifacts and PET-CT misalignment
Source: Eur J Nucl Med Mol Imaging. 2024 Aug 13;52(1):62–73. doi: 10.1007/s00259-024-06872-x (PMC11599311; doi:10.1007/s00259-024-06872-x)
Supplement: Supplementary file 1 — Supplementary Material 1 [file 259_2024_6872_MOESM1_ESM.pdf]

European Journal of Nuclear Medicine and Molecular Imaging:

Deep learning-aided respiratory motion compensation in PET/CT: Addressing motion induced resolution loss, attenuation correction artifacts and PET-CT misalignment

Yihuan Lu[1#], Fei Kang[2#], Duo Zhang[1], Yue Li[1], Hao Liu[1], Chen Sun[1], Hao Zeng[1], Lei Shi[1], Yumo Zhao[1], Jing Wang[2]

[1] United Imaging Healthcare, Shanghai, China

[2] Department of Nuclear Medicine, Xijing Hospital, Fourth Military Medical University, Xi'an, China.

# These authors contributed equally to this article.

Co-first authors:

Yihuan Lu, United Imaging Healthcare, No. 2258 Chengbei Road, Shanghai, China, E-mail: yihuan.lu@united-imaging.com.

Fei Kang, Department of Nuclear Medicine, Xijing Hospital, 127 West Changle Road, Xi'an, China, 710032. E-mail: fmmukf@qq.com.

Correspondence contacts:

Yihuan Lu, United Imaging Healthcare, No. 2258 Chengbei Road, Shanghai, China, 201807. E-mail: yihuan.lu@united-imaging.com.

Jing Wang, Department of Nuclear Medicine, Xijing Hospital, 127 West Changle Road, Xi'an, China, 710032. E-mail: 13909245902@163.com.

Supplemental Material

Body cavity segmentation

The neural network used for body cavity segmentation (CNN_{cavity}) is a customized 3D U-Net with residual units, trained on 160 whole-body CT volume images. These 160 CT images were randomly selected from the training dataset, with manual body cavity segmentations by experienced physicians serving as the training labels. To ensure the accuracy of the segmentation results, two physicians independently performed the body cavity segmentation. A third physician then evaluated these results and selected the one with the better performance as the final segmentation label. Before training, the CT images were resampled to a voxel size of 6×6×6 mm^3 and underwent image intensity normalization using the following formula:

$$I_{i,norm} = \frac{I_i - I_{min}}{I_{max} - I_{min}}$$

Where I_i is the original HU value of voxel i; I_{max} and I_{min} are the maximum and minimum values of the entire image. Data augmentation was performed by resizing the original image volume by 30% to 100% with a 20% probability, using uniform distribution sampling. The learning rate was set to 10^{-4} and Adam optimizer was used. Each training batch contains 20 randomly sampled image patches, which consists of 96×96×96 voxels. The network was trained for 8,000 epochs with batch normalization.

To evaluate the performance of a body cavity segmentation neural network, we randomly selected a cohort of 80 CT images from the training set, using their corresponding manually segmented labels by physicians as the reference standard. The Dice coefficient and Hausdorff distance (HD) were employed as metrics to compare the differences between the body cavity segmentations mask produced by the neural network and the reference labels. The Dice coefficient measures the overall overlap ratio between the gold-standard and the target segmentations, while the Hausdorff distance quantifies the largest edge mismatch between the two masks.

$$\text{Dice} = \frac{2(Y \cap \hat{Y})}{(Y \cup \hat{Y}) + \varepsilon}$$

where ε was set to 10^{-5}. Y and $\hat{Y}$ represent the label mask and the inference mask, respectively.

$$\text{HD} = \max\{\text{h}(Y,\hat{Y}), \text{h}(\hat{Y},Y)\}, \text{ where}$$

$$\text{h}(Y,\hat{Y}) = \max_{y \in Y}\{\min_{x \in \hat{Y}} ||x - y||\} \text{ and } \text{h}(\hat{Y},Y) = \max_{x \in \hat{Y}}\{\min_{y \in Y} ||x - y||\},$$

where $||x\text{-}y||$ represents the Euclidean distance between the voxels x and y.

The neural network achieved accurate segmentation of the body cavities across all 80 cases. Supplemental Fig. 1 shows the Dice coefficient and HD results for the testing datasets. The average Dice value of the 80 testing cases is 97.2 ± 1.2% while the average HD value is 15.8 ± 5.7 mm. In addition, two sampled CT images are also shown in

Supplemental Fig. 2, with blue mask indicating the reference label of the body cavity and red mask indicating the segmented result produced by the neural network.

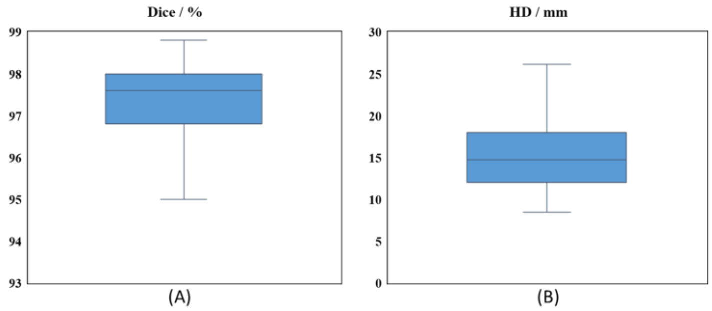

Supplemental Fig. 1. Box-plot of the Dice coefficient values (A) and the HD values (B) from the 80 testing datasets.

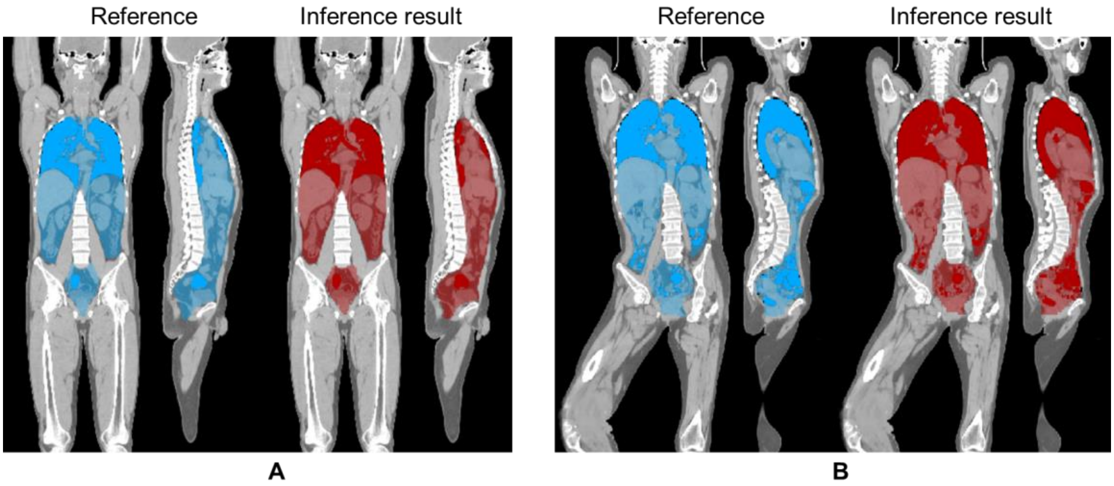

Supplemental Fig. 2. Two sampled CT images with blue mask indicating the reference label of the body cavity and red mask indicating the segmented result produced by the neural network. The left image (A) illustrates a case with relatively good segmentation results (Dice coefficient of 98.8% and HD of 8.5 mm), while the right image (B) depicts a case with relatively poorer results (Dice coefficient of 96.8% and HD of 17.0 mm).

COD based signal detection

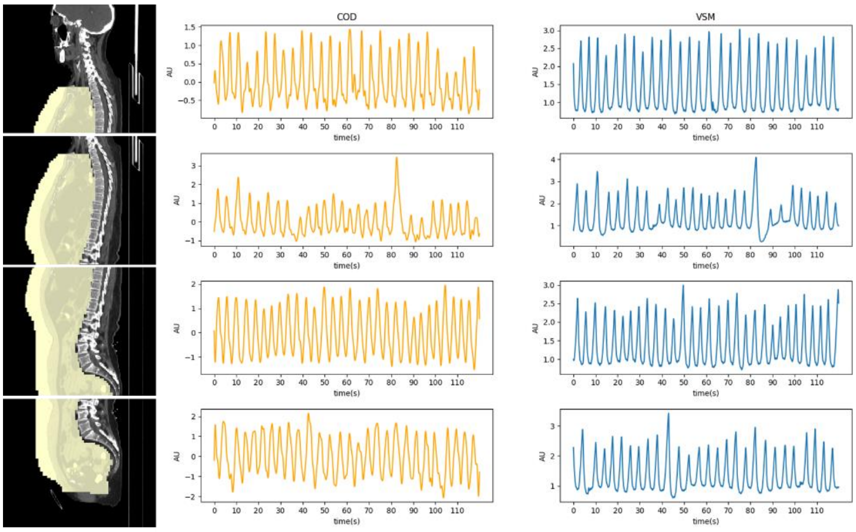

Supplemental Fig. 3 Example of COD vs. VSM signal for respiratory detection at four different beds. Yellow mask indicates the region to extract respiratory signal using the COD method.

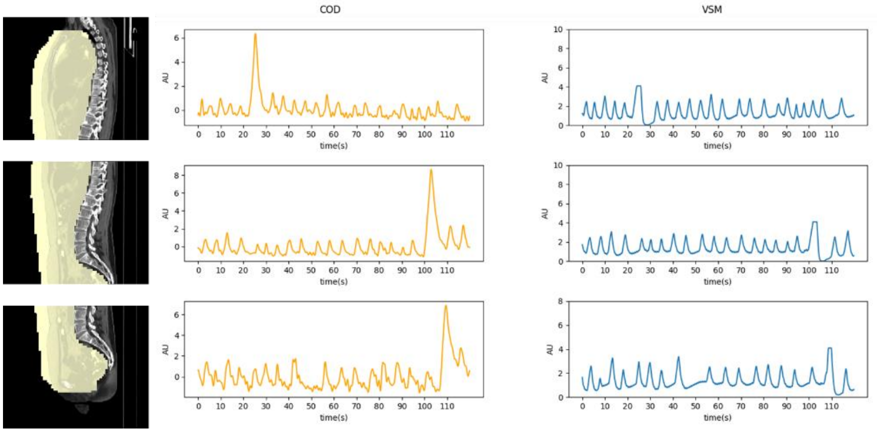

Supplemental Fig. 4 Example of signal saturation in VSM while not in COD at four different beds. Yellow mask indicates the region to extract respiratory signal using the COD method.

AI-based attenuation correction

The deep learning (DL) network designed to predict the gated attenuation map (CNN_{mu}) is a customized 3D U-net with residual units. It has been trained using PET images reconstructed without attenuation correction (NAC) and the attenuation maps derived from CT images (μ-CT) for 638 cases in the training set. Specifically, the raw PET data were divided into four gates based on respiratory signals, and NAC PET data were reconstructed for each gate to obtain four gated PET NAC images. The CT images were acquired while the patients were instructed to breathe shallowly, theoretically allowing for good alignment with one of the gated PET images. Through visual assessment,

we identified the gated PET image that best matched the CT image. This pair of NAC PET and CT-derived attenuation maps μ-CT was then used as the input and target, respectively, for training the DL network.

The intensity of the gated NAC image was rescaled to 0.0 - 1.0 before being used for network training. All training inputs and label μ-CT were resampled to a voxel size of 2.4 × 2.4 × 2.94 mm³. Data augmentation was performed by resizing (50% probability) the original image volume from 100% to 130% with uniform distribution sampling. The learning rate was set at 10^{-3}, and an Adam optimizer was used. Each training batch contained 16 randomly sampled image patches, each consisting of 64×64×32 voxels. Resolution reduction was performed using 2 × 2 × 2 convolution kernels with a stride of 2. A total of 20,000 epochs were used in training with batch normalization.

Application of uRMC on PET/CT images with CT artifacts

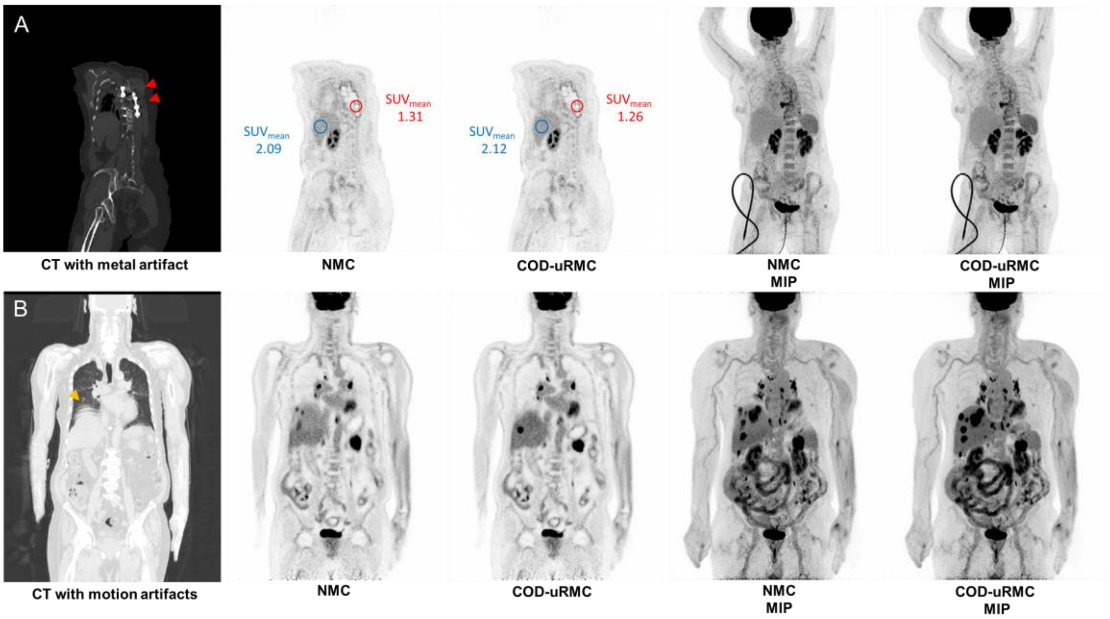

Supplemental Fig. 5 Two sampled cases to show the result of utilizing uRMC on PET/CT images in the presence of metal artifacts (A) or respiratory motion artifacts (B) in the CT images. The red arrows in (A) indicate the region where mental artifacts are present and yellow arrow in (B) point to the region where respiratory motion artifacts are present.

PET/CT mis-registration caused AC artifacts in respiratory gated PET images

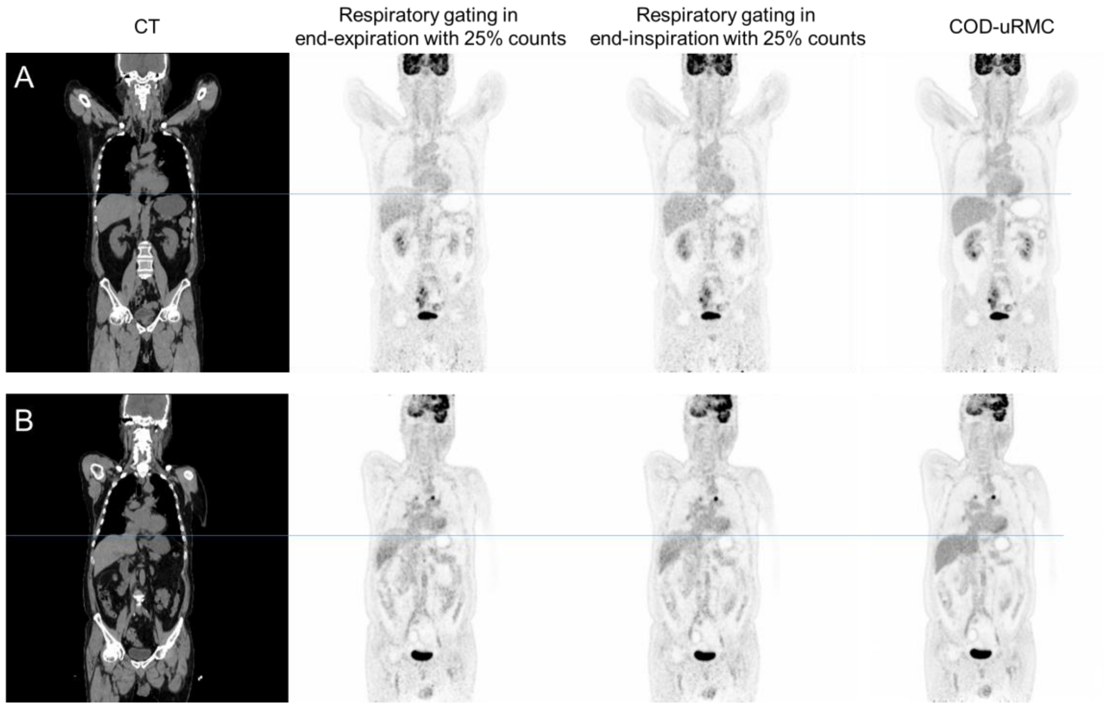

Supplemental Fig. 6 Two sampled cases to show the PET/CT mis-registration caused AC artifacts in the respiratory-gated PET images. (A) CT was acquired during shallow breathing which could match with the respiratory gated PET in end-inspiration well while severe AC mismatch artifacts can be noticed in respiratory gated PET in end-expiration. (B) CT was acquired with deep-inspiration, lead to AC artifacts in both the end-inspiration and end-expiration gated PET images when using the CT for attenuation correction. Our proposed uRMC approach resolves the PET/CT mis-registration issues, effectively addressing the AC mismatch artifacts.
